# Supplementary figures and images for: Optimized Detection of Acute MHV68 Infection With a Reporter System Identifies Large Peritoneal Macrophages as a Dominant Target of Primary Infection
Source: Front Microbiol. 2021 Mar 9;12:656979. doi: 10.3389/fmicb.2021.656979 (PMC7985543; doi:10.3389/fmicb.2021.656979)

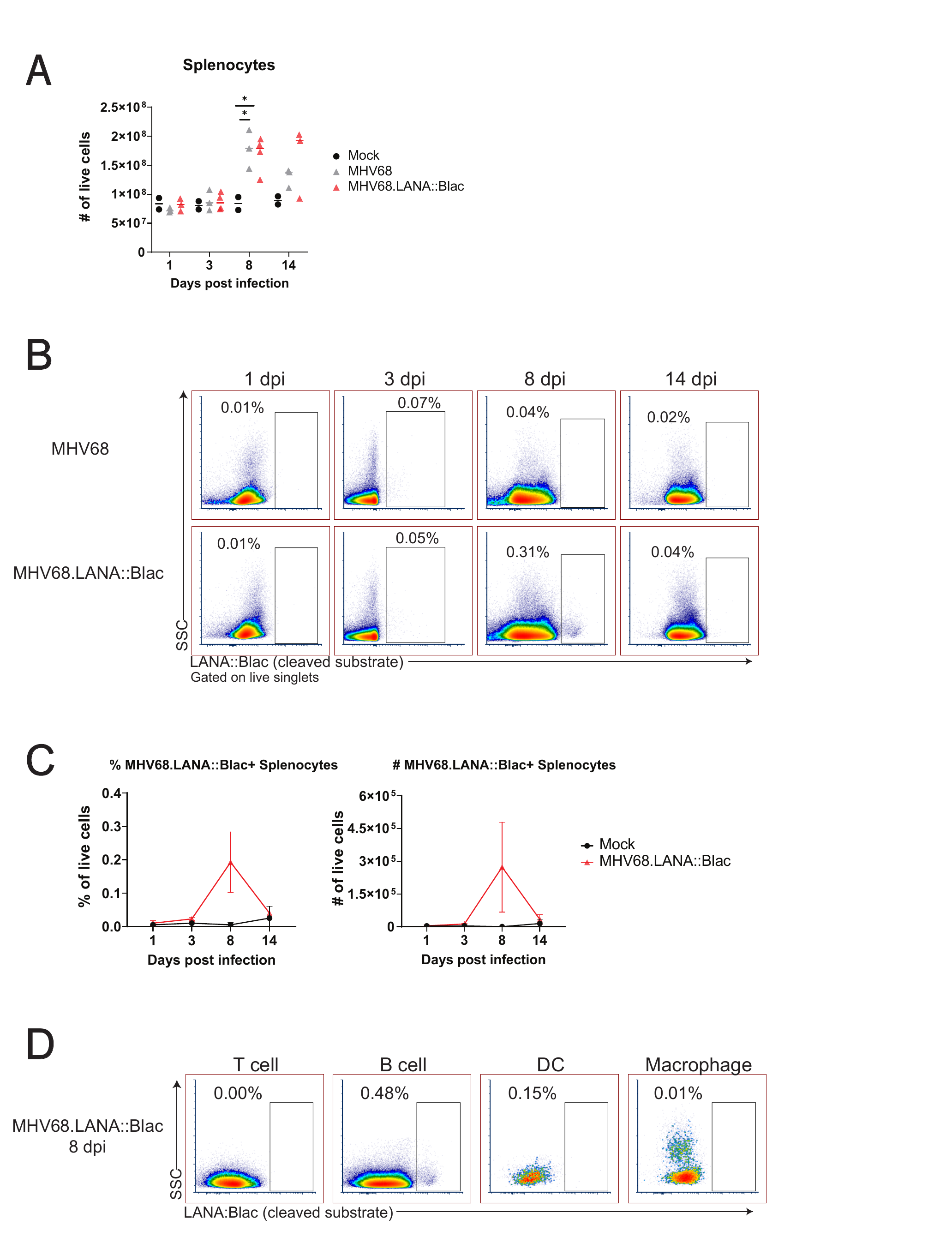

Supplement: Supplementary Figure 1 — MHV68.LANA:βlac is detectable in B cells at 8 days post infection in the spleen. C57BL/6 mice were infected i.p. with 1 million PFUs of WT MHV68 or MHV68.LANA:βlac. At 1, 3, 8, and 14 dpi, mice were sacrificed and spleens were harvested, counted, and stained with CCF2-AM, viability, and surface markers. (A) Viable cell counts of harvested spleens in mock, MHV68 or MHV68.LANA:βlac infected mice. (B) Dot plots showing the frequency of splenocytes positive for cleaved CCF2. LANA:βlac signal is enriched above background (defined by WT MHV68 infection) at 8 dpi. (C) Quantification of the mean frequency and number of LANA:βlac+ cells shown in (B). (D) Dot plots showing percentage of T cells, B cells, dendritic cells, and macrophages that are positive for cleaved CCF2. B cells are the only population with cleaved CCF2 signal, with about 0.5% of cells staining positive. Data representative of three experiments with 2–3 mice per condition per experiment. [file Image_1.TIFF]

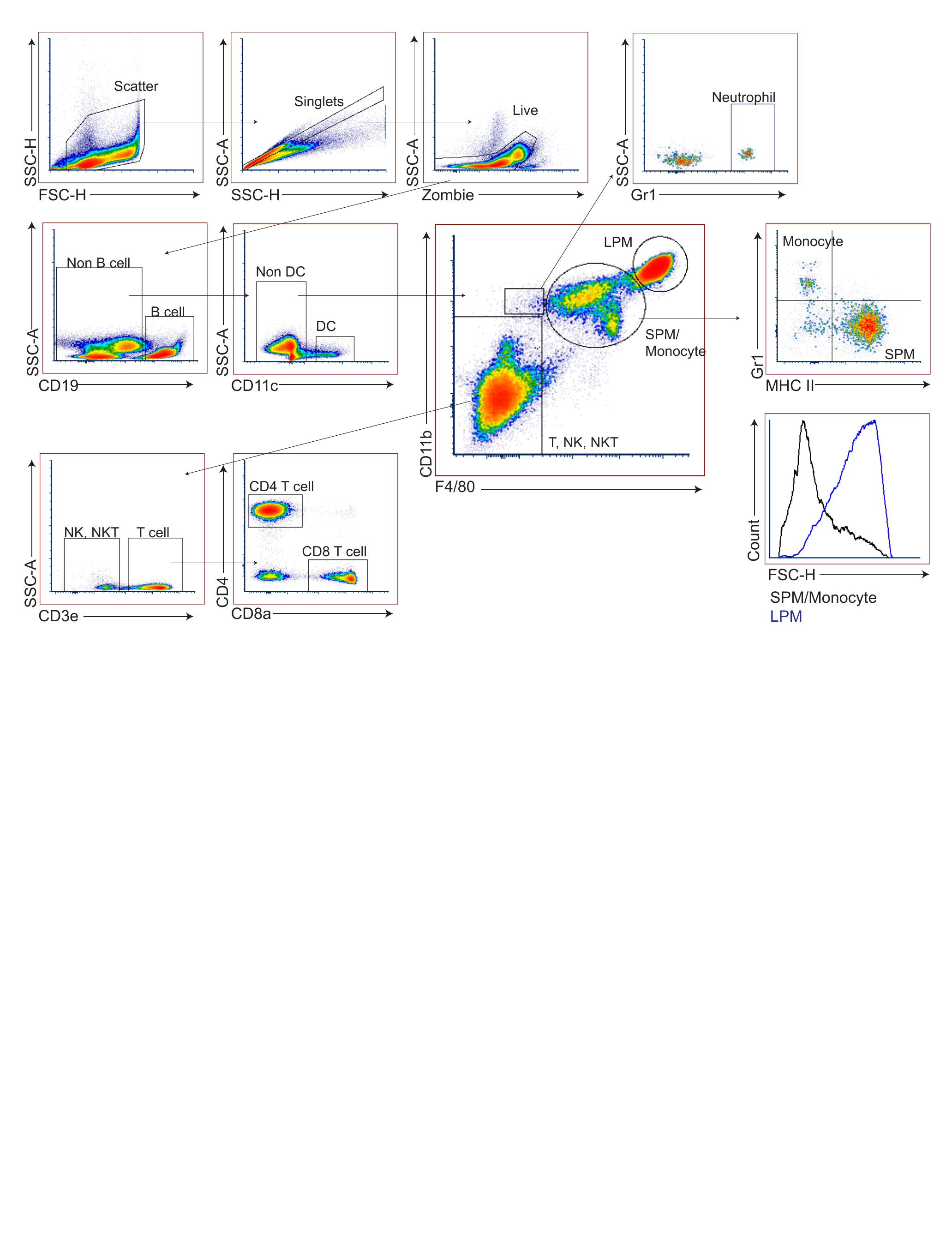

Supplement: Supplementary Figure 2 — Gating strategy for analysis of spectral flow cytometry data. Representative gating scheme adopted from Ghosn et al. (2010). Sample derived from PerCs of a mock-infected mouse. FSC histogram denotes size difference between SPM/monocyte and LPM populations. [file Image_2.TIFF]
